# Knowledge and Awareness of Human Papillomavirus Vaccination in an Urbanized Village in Delhi

**DOI:** 10.7759/cureus.84512

**Published:** 2025-05-20

**Authors:** Swaleha Furqan, Anubhav Mondal, Nidhi Singh, Monu Singh, Richa Kapoor

**Affiliations:** 1 Community Medicine, Vardhman Mahavir Medical College and Safdarjung Hospital, New Delhi, IND

**Keywords:** awareness, cervical cancer, hpv, hpv vaccination, knowledge

## Abstract

Introduction: Cervical cancer is one of the major health problems and is a leading cause of death among women worldwide. Awareness regarding the prevention strategies against cervical cancer plays a major role in reducing its burden. Hence, the current study was planned with the objective to assess the level of awareness and knowledge regarding the human papillomavirus (HPV) vaccine.

Materials and methods: A cross-sectional study was conducted to include 405 participants using simple random sampling from an urbanized village of Delhi from March 2023 to April 2023. Data was collected using a pre-designed, semi-structured questionnaire. Tests of association, such as the chi-square test and its modifications, were used to determine statistical significance. P < 0.05 was taken as statistically significant.

Results: Most of the participants (95.6%) were not aware of the HPV vaccine. Only 1.75% correctly knew who needs the HPV vaccine, and 0.2% correctly knew at what age the vaccine is to be given. Out of those who knew about the HPV vaccine, 83% of study participants were willing to take the vaccine. No statistically significant association was found with the sociodemographic variables.

Conclusion: There was a considerable lack of awareness and knowledge regarding the HPV vaccine among the residents of the urbanized village. Thus, it presents an opportunity that should be utilized to reach out and develop community awareness to encourage acceptance of the HPV vaccine.

## Introduction

Cervical cancer is the fourth most common cancer among women worldwide, with approximately 660,000 new cases and 350,000 deaths reported globally in 2022 [1,2]. A significant proportion of these cases occur in low- and middle-income countries, with India, China, and Brazil accounting for the majority of cervical cancer burden [3]. According to the Cervical Cancer India 2021 Country Profile, the crude incidence of cervical cancer in India was 18.7 per 100,000 women in 2020, and approximately 45,300 women died from the disease in 2019 [4]. The cumulative risk of cervical cancer between the ages of 0-74 years was estimated to be 2% [4].

Persistent infection with high-risk strains of human papillomavirus (HPV), particularly types 16 and 18, is the primary etiological factor for cervical cancer [5]. HPV is primarily transmitted through sexual contact, and preventive measures such as vaccination against HPV and routine screening have proven to be highly effective in reducing disease burden. HPV vaccination is a safe and effective way to prevent infections caused by HPV, which can lead to cervical, anal, and other cancers. It is recommended for adolescents, ideally before the onset of sexual activity, to provide maximum protection.

HPV vaccines, if administered prior to exposure to high-risk strains, significantly reduce the incidence of cervical intraepithelial neoplasia and subsequent cervical cancer. Developed countries have successfully implemented cytology-based screening (e.g., Pap smear), while low-resource settings have adopted alternative screening methods such as visual inspection with acetic acid (VIA) and Lugol’s iodine (VILI). By early screening, cervical cancer is largely preventable.

High-risk HPV infections in Indian males contribute to cancers of the penis, anus, and oropharynx, presenting an underrecognized public health challenge [6]. Studies indicate a significant proportion of Indian men carry oncogenic HPV strains, with certain types detected in nearly half of penile cancer cases, which account for a small but notable percentage of male malignancies nationally. Immunocompromised individuals, particularly those with HIV, face substantially higher risks for these HPV-associated cancers [7].

Men play a crucial role in HPV transmission dynamics, serving as carriers who can unknowingly spread the virus to female partners. This transmission pathway significantly contributes to India's high cervical cancer burden among women. Vaccinating males would create a dual benefit - directly protecting them from HPV-related cancers while simultaneously reducing viral circulation in the population. 

Despite global efforts, awareness and acceptance of HPV vaccination remain suboptimal, particularly in community settings where educational outreach is limited. Most previous studies have focused on women, often in academic institutions, neglecting broader community perspectives, including both genders [8-10]. Given the role of both men and women in HPV transmission and disease burden, this study aimed to assess the level of awareness and knowledge regarding HPV vaccination among male and female residents of an urbanized village in Delhi.

## Materials and methods

This community-based cross-sectional study was conducted from March to April 2023 in Aliganj, an urbanized village in South Delhi, falling under the Kotla Mubarakpur ward, with a total population of 6,139. There were 2,848 households. The participating population included male and female residents aged 15-49 years.

Sample size

On the basis of a study conducted by Singh J et al. in the year 2018, the prevalence of knowledge regarding the HPV vaccine was found to be 40.2% among participants of similar study settings [11].

Taking this as prevalence, the sample size was calculated: 1) P = 40.2% (prevalence), 2) Q = 59.8% (1-P), 3) taking L = 5% (absolute error).

After putting these values in the Cochran's formula: (Zα/2) 2 PQ/ L 2, the sample size came out to be 369.

Taking a non-response rate of 10%, the total sample size was computed to be 405.

Sampling procedure

A comprehensive household list of Aliganj was generated and used as the sampling frame. The list was entered in MS Excel (Microsoft Corporation, USA), and 405 households were randomly selected. One individual, meeting the inclusion criteria, was included from each household in the study. If more than one eligible individual was present in a household, the KISH grid method was employed to randomly select one participant. The questionnaire was administered after explaining the study objectives and obtaining written informed consent.

Data collection tool

A pre-tested, semi-structured, interviewer-administered questionnaire was used to collect information on sociodemographic characteristics, awareness, knowledge, and attitudes regarding HPV vaccination. Participants who did not respond after three visits were excluded.

Operational definitions

"Urbanized village" is an unplanned settlement beyond formal jurisdiction, often lacking proper water, drainage, and sanitation infrastructure [12]. "Migrant" is an individual enumerated at a location different from their place of birth or last residence [13].

Statistical analysis

Data were analyzed using IBM SPSS Statistics for Windows, version 21.0 (released 2012, IBM Corp., Armonk, NY). Descriptive statistics were presented as means, standard deviations, and proportions. Associations between awareness and sociodemographic variables were evaluated using Chi-square and Fisher’s exact tests. A p-value <0.05 was considered statistically significant.

## Results

Of the 405 participants, 175 (43%) were male and 230 (57%) were female. The mean age was 31.9 ± 9.0 years. Most were married (72.8%) and migrants (76.7%). Educational status varied, with 44(10.8%) being illiterate and 113 (28%) educated up to middle school. Some form of employment was reported by 164 (40.2%), while 157 (38.8%) were homemakers, 72 (17.8%) students, and 12 (3%) unemployed. Most belonged to nuclear families (371, 91.6%) and were Hindus (375, 91.6%) by religion. Based on the revised modified Kuppuswamy socioeconomic scale (2022), 167 (41.2%) were from the upper middle class, followed by 142 (35%) from the middle class (Table 1).

**Table 1:** Distribution of the study participants according to the sociodemographic characteristics (N = 405)

S. No.	Participant characteristics	Frequency (N)	Percentage (%)
1.	Gender		
	Male	175	43
	Female	230	57
2.	Education status		
	Illiterate	46	11.2
	Primary school certificate	41	10.1
	Middle school certificate	113	28
	High school certificate	106	26.2
	Intermediate/Diploma	28	7
	Graduate	71	17.5
3.	Age		
	<34 years	230	56.8
	>35 years	175	43.2
4.	Religion		
	Hindu	375	92.6
	Muslim	21	5.1
	Christian/others	9	2.1
5.	Type of family		
	Joint	34	8.3
	Nuclear	371	91.6
6.	Migrant status		
	Yes	311	76.7
	No	94	23.2
7.	Marital status		
	Married	295	72.8
	Unmarried	103	25.4
	Widowed/separated /divorced	7	1.72
8.	Occupation		
	Unemployed	12	3
	Homemaker	157	38.8
	Student	72	17.8
	Unskilled	48	11.9
	Semiskilled	53	13.1
	Skilled	41	10.1
	Semiprofessional	3	0.7
	Professional	1	0.2
	Clerical	18	4.4
9.	Socioeconomic class		
	Upper	70	17.3
	Upper middle	167	41.3
	Middle	142	35
	Lower middle	24	5.9
	Lower	2	0.5

Only 18 (4.4%) participants were aware of the HPV vaccine. Among these, just seven (1.7%) study participants correctly identified the target population, and one (0.2%) study participant knew the correct age for vaccination. Only seven (1.75%) participants were aware of government HPV vaccination drives. Three (0.7%) study participants were aware of the indigenously produced “Cervavac” vaccine. Only two (0.5%) participants knew of any nearby vaccination center (Table 2).

**Table 2:** Distribution of the study participants according to their knowledge regarding HPV vaccination (N = 405).

Questions	Responses	Frequency (N)	Percentage (%)
Do you know who needs the HPV vaccine			
	Males	0	0
	Females	5	1.2
	Both	7	1.7
	Don't know	393	97
Do you know at what age the HPV vaccine is given			
	Knows correctly	1	0.2
	Knows incorrectly	5	1.2
	Don't know	399	98.6
Are you aware of the HPV vaccination drive initiated by the Delhi government?			
	Yes	7	1.7
	No	398	98.3
Do you know about the endogenously manufactured HPV vaccine CERVAVAC?			
	Yes	3	0.7
	No	402	99.3
Have you heard about any other brand of HPV vaccine?			
	Knows correctly	1	0.2
	Don't know	404	99.8
Do you know the nearest centre where you can be vaccinated?			
	Yes	2	0.5
	No	403	99.5

Among those aware, 15 (83%) were willing to receive the vaccine themselves, 15 (83%) supported vaccinating their partner, and 17 (93%) were willing to vaccinate their children. However, only six (33.3%) were willing to pay for the vaccine, while nine (50%) declined and three (16.7%) were unsure. However, 16 (88.9%) participants were willing when the vaccine was offered free of cost. Notably, 16 (88.9%) participants desired more information about the vaccine (Table 3).

**Table 3:** Distribution of study participants according to attitude regarding HPV vaccination (n = 18)

Attitude	Agree (n,%)	Disagree (n,%)	Can't say (n,%)
1. I/my parents would pay for the vaccine.	6 (33.3%)	9 (50%)	3 (16.7%)
2. I would get the vaccine if it were free of cost.	16 (88.9%)	2 (11.1%)	0 (0%)
3. It's not necessary for me to get vaccinated.	2 (11.1%)	14 (77.8%)	2 (11.1%)
4. I wish to get more information on the HPV vaccine.	16 (88.9%)	2 (11.1%)	0 (0%)

No statistically significant association was found between HPV vaccine awareness and sociodemographic variables (p > 0.05)(Table 4).

**Table 4:** Distribution of the study participants according to awareness to get vaccinated (N = 405) The chi-square test was used to test statistical significance. # Fischer exact modification was used. A p-value greater than 0.05 was taken as significant.

Variable	Unaware (N,%)	Aware (N, %)	Total (N, %)	χ² value	P-value
Gender				3.52	0.06#
Male	171 (42.2%)	14 (3.4%)	230 (56.8%)		
Female	216 (53.3%)	4 (0.9%)	220 (43.2%)		
Education				2.29	0.130#
Illiterate	44 (10.9%)	0 (0%)	44 (10.9%)		
Literate	343 (84.7%)	18 (4.4%)	361 (89.1%)		
Age				0.16	0.689
<34	218 (53.8%)	11 (2.7%)	229 (56.5%)		
>35	169 (41.7%)	7 (1.7%)	176 (43.5%)		
Family type				0.18	0.671#
Joint	32 (7.9%)	2 (0.5%)	34 (8.4%)		
Nuclear	355 (87.6%)	16 (3.9%)	371 (91.6%)		
Migrant status				1.12	0.29
Yes	301 (74.3%)	10 (2.7%)	311 (76.8%)		
No	86 (21.2%)	8 (1.9%)	94 (23.2%)		
Marital status				2.83	0.092
Married	285 (70.4%)	10 (2.7%)	295 (72.8%)		
Others	102 (25.2%)	8 (1.9%)	110 (27.2%)		
Occupation				0.40	0.527
Unemployed	229 (56.5%)	12 (2.9%)	241 (59.5%)		
Employed	158 (39.0%)	6 (1.5%)	164 (40.5%)		

## Discussion

The findings revealed alarmingly low awareness (4.4%) regarding HPV vaccination among residents of an urbanized village in Delhi, despite high levels of willingness to receive the vaccine if made accessible and affordable. These results are consistent with those of Rao BA et al., who reported similar awareness levels in urban low-resource settings [14]. The contrast with developed countries, where awareness levels are substantially higher (e.g., 67.1% in the U.S. as reported by Thompson EL et al. [15], underscores disparities in health literacy and outreach efforts. This further highlights the importance of health promotional activities in order to create health need for the vaccine.

Prior studies in India, such as those by Singh J et al. and Rehman A et al., also report low awareness (18-22%), although higher than observed in our study [5,11]. The higher proportion of participants with educational attainment below high school (48%) may partly explain this gap. Furthermore, unlike many prior studies focusing on women alone, our study included both genders, thereby offering a more comprehensive community perspective.

Awareness is the first step toward developing a perceived health need, which is essential for the uptake of preventive services like HPV vaccination. Creating awareness can stimulate health-seeking behavior and community-level demand. This is particularly relevant in light of India’s announcement in the Union Budget 2024 regarding the encouragement for cervical cancer immunization. Without sufficient public awareness and health education, such efforts risk suboptimal coverage.

In the present study, awareness regarding the HPV vaccine was found to be higher among males. This disparity may be attributed to greater access to health information, digital resources, and educational opportunities among men. Sociocultural norms, stigma surrounding female reproductive health, and male-dominated health decision-making further contribute to lower awareness among women [16].

In our study, 83% of the people aware of the HPV vaccine were willing to take the vaccine. This is similar to the findings of the study by Chowdhury et al. [17], although there was a difference in the study population. Chowdhury et al. conducted the study among medical students, while ours was a community-based study. This reflects the adaptability of the study population in case of vaccine introduction.

Only 20% of the participants were initially willing to pay for the HPV vaccine; however, the proportion increased significantly when the vaccine was offered free of cost. This underscores cost as an important barrier to vaccine uptake, particularly in low-resource settings. Financial constraints, limited insurance coverage, and low perceived risk may deter individuals from paying out-of-pocket for preventive health services [18].

Although the state government has initiated school-based opportunistic vaccination programs since 2016, our study found only 1.75% awareness of such initiatives. In addition, awareness about “Cervavac,” the indigenous quadrivalent HPV vaccine manufactured by the Serum Institute of India, was minimal (0.7%), reflecting gaps in both communication and public engagement. A study conducted by Kadian et al. [19] also revealed similar results, with only 4.3% having good knowledge about the HPV vaccine.

This study is among the few community-based investigations evaluating awareness of HPV vaccination among the general population, including both men and women of reproductive age. Its strength lies in its representativeness and robust sampling methodology.

However, being a cross-sectional study, it is limited in its ability to establish causal relationships. Social desirability bias may have influenced responses, particularly regarding willingness to vaccinate. Furthermore, the questionnaire did not explore in-depth factors such as healthcare access, cultural beliefs, or perceived barriers, which may also influence vaccine uptake.

## Conclusions

This study highlights critically low awareness and knowledge regarding HPV vaccination among residents of an urbanized village in Delhi. Despite this, there is considerable willingness to accept the vaccine, particularly if offered free of cost. The findings suggest that proactive health promotion and targeted educational campaigns are urgently needed to enhance community-level demand. As the Government of India prepares for a national rollout of HPV vaccination, increasing awareness through strategic health communication is vital to ensure the success of the initiative. Efforts should focus on improving literacy about cervical cancer prevention, demystifying vaccination, and addressing cost-related and informational barriers.

## Appendices

**Table 5:** Questionnaire

S. No.	Question
	Name:
	Age:
	Sex: a. male_____ b.female_____ c.other_____
	Religion: Hindu_____ Muslim_____ Christian_____ Other_____
	Educational status: Profession/honours_____ Graduate____ Intermediate or diploma____ High school certificate____ Middle school certificate____ Primary school certificate____ Illiterate
	Marital status: Unmarried_____ Married_____ Divorced______ Separated_____ Widowed_____
	Occupation:
	Family income per month:
	Number of family members:
	Number of children: Age____ Sex_____ Age____ Sex_____
	Type of family- joint_____ nuclear_____
	Are you a migrant? Yes____ No____
	Have you heard about the human papilloma virus? Yes___ No___
	Do you know that HPV is different from HIV? Yes___ No___ Don’t know___
	Do you know some diseases caused by HPV? Correct____ Incorrect____ Don’t know___
	Do you know that HPV causes cervical cancer? Yes_____ No______ Don’t know______
	Have you heard about the HPV vaccine/cervical cancer vaccine? Yes____ No_____
	Do you know who needs HPV vaccine? Male____ Female____ Both____
	Do you know at what age the HPV vaccine is given? Correct_____ Incorrect____ Don’t know_____
	Are you aware of the HPV vaccination drive initiated by the Delhi Government? Yes____ No____
	Do you know about the indigenously manufactured HPV vaccine CERVAVAC? Yes_____ No_____
	Do you know the nearest centre where you can be vaccinated? Yes____ No____ If yes, where? ____
	How did you come to know about HPV vaccine? TV_____ Internet______ Doctor_______ Studies_______ Know someone with cervical cancer_______ Friends/Relatives/Neighbours_______ Newspaper________ Anganwadi worker_______ Other_______
	Are you willing to take HPV vaccine? Yes_____ No_____
	If no, why? Don’t know about HPV vaccine_______ Don’t know where it is available_________ High cost of vaccine________ Do not consider myself to be at risk of HPV_______ Other________
	Do you consider useful vaccinating your partner against HPV? Yes______ No______
	Do you consider vaccinating your children against HPV? Yes____ No_____
	If yes, which child? Male child_____ Female child_____ All children_____
	I/my parents would pay for the vaccine. Agree__ Disagree__ Can’t say__
	I would get the vaccine if it were free. Agree__ Disagree__ Can’t say__
	It’s not necessary for me to get vaccinated. Agree__ Disagree__ Can’t say__
	I wish to get more information on the HPV vaccine. Agree__ Disagree__ Can’t say__
	Are you vaccinated against HPV? Yes______ No______
	Is any of the family members vaccinated? Yes______ No______
